# Ceramide Kinase Is Upregulated in Metastatic Breast Cancer Cells and Contributes to Migration and Invasion by Activation of PI 3-Kinase and Akt

**DOI:** 10.3390/ijms21041396

**Published:** 2020-02-19

**Authors:** Stephanie Schwalm, Martin Erhardt, Isolde Römer, Josef Pfeilschifter, Uwe Zangemeister-Wittke, Andrea Huwiler

**Affiliations:** 1 Institute of Pharmacology, University of Bern, Inselspital, INO-F, CH-3010 Bern, Switzerland; s.schwalm@med.uni-frankfurt.de (S.S.); martin.erhardt@pki.unibe.ch (M.E.); 2 Institute of General Pharmacology and Toxicology, University Hospital Frankfurt am Main, Goethe-University, Theodor-Stern Kai 7, D-60590 Frankfurt am Main, Germany; I.Roemer@em.uni-frankfurt.de (I.R.); Pfeilschifter@em.uni-frankfurt.de (J.P.)

**Keywords:** ceramide kinase, metastatic breast cancer cells, migration and invasion

## Abstract

Ceramide kinase (CerK) is a lipid kinase that converts the proapoptotic ceramide to ceramide 1-phosphate, which has been proposed to have pro-malignant properties and regulate cell responses such as proliferation, migration, and inflammation. We used the parental human breast cancer cell line MDA-MB-231 and two single cell progenies derived from lung and bone metastasis upon injection of the parental cells into immuno-deficient mice. The lung and the bone metastatic cell lines showed a marked upregulation of CerK mRNA and activity when compared to the parental cell line. The metastatic cells also had increased migratory and invasive activity, which was dose-dependently reduced by the selective CerK inhibitor NVP-231. A similar reduction of migration was seen when CerK was stably downregulated with small hairpin RNA (shRNA). Conversely, overexpression of CerK in parental MDA-MB-231 cells enhanced migration, and this effect was also observed in the non-metastatic cell line MCF7 upon CerK overexpression. On the molecular level, CerK overexpression increased the activation of protein kinase Akt. The increased migration of CerK overexpressing cells was mitigated by the CerK inhibitor NVP-231, by inhibition of the phosphoinositide 3-kinase (PI3K)/Akt pathway and the Rho kinase, but not by inhibition of the classical extracellular signal-regulated kinase (ERK) pathway. Altogether, our data demonstrate for the first time that CerK promotes migration and invasion of metastatic breast cancer cells and that targeting of CerK has potential to counteract metastasis in breast cancer.

## 1. Introduction

Metastatic breast cancer is a devastating disease with a poor prognosis [1,2]. There is growing evidence that bioactive lipid molecules are implicated in malignant progression by regulating proliferative, migratory, and invasive behavior of cancer cells.

Ceramide and sphingosine 1-phosphate (S1P) are key bioactive lipid molecules that constitute the cellular sphingolipid rheostat and determine a cell’s fate either to live or to die [3]. Numerous studies have highlighted the pro-apoptotic and the anti-proliferative function of ceramide in many cell types, whereas S1P is considered a counter-molecule to ceramide and exerts opposite effects, such as promoting cell proliferation and survival [4,5,6,7]. However, several new bioactive sphingolipid molecules have emerged from recent studies, and this view of the ceramide/S1P rheostat function is oversimplified. One molecule that has recently attracted much attention is ceramide 1-phosphate (C1P), which is generated from ceramide by the action of ceramide kinase (CerK) [8,9]. Although the detailed cellular mechanisms of action and the direct targets of C1P are still largely unclear, targeting of CerK by either genetic approaches, such as siRNA and genetic knockout in mice, or pharmacologically with, e.g., catalytic inhibitors, has unveiled a number of physiological and pathophysiological functions [9]. These include regulation of intracellular vesicle formation, fusion and release [10,11,12], as well as cell proliferation and migration [8,13,14], and inflammation [9,15].

The human CerK gene translates into a protein of 537 amino acids with a predicted molecular size of 59 kDa, which shows the highest homology to the diacylglycerol kinase and the sphingosine kinase 1 [16]. The subcellular localization of CerK is variable and includes Golgi, nucleus, cytoplasm, plasma membrane, and even mitochondria [17,18,19]. As amino acid sequence analysis unveiled a putative nuclear import signal at the N-terminal region and a nuclear export signal at its C-terminal end, the hypothesis of a nucleocytoplasmic shuttling was suggested to have relevance for its regulation and function [17]. On the opposite side, a N-terminal pleckstrin homology (PH) domain of CerK interacts with high affinity with the lipid phosphatidylinositol 4,5-bisphosphate, and this mediates rapid translocation of the enzyme to the plasma membrane [18]. In addition, a C1P-transfer protein (CPTP, GLTPD1) was identified that transports Golgi-localized C1P to the plasma membrane and may participate in regulating subcellular localization and action of C1P, such as in inflammation and autophagy [20].

We previously suggested a prominent role of CerK in cell cycle and mitosis regulation in cancer cells. By using a potent and selective CerK inhibitor, we showed that, in breast and lung cancer cell lines, inhibition of CerK reduced viability, DNA synthesis, and colony formation. Cells were arrested in the M phase of the cell cycle and subsequently underwent apoptosis [14]. Recently, CerK has also been suggested to increase malignancy, as it was shown that, in pancreatic cancer cells, extracellular C1P, but also overexpression of CerK and intracellularly generated C1P, increased migration and invasion. Mechanistically, this process was dependent on phosphoinositide 3-kinase (PI3K), mTOR, Akt, and RhoA [13]. In addition, CerK expression was found to be higher in estrogen receptor (ER)-negative than in ER-positive breast cancer, and within the ER-negative subgroup of patients, those with highest CerK expression had the worst prognosis and the shortest survival [21]. Furthermore, a study included 2200 breast cancer patients in a gene expression analysis of CerK, and found an association of increased CerK expression with increased risk of cancer recurrence [22].

Here, we used the triple negative human breast cancer cell line MDA-MB-231 and two sublines established from its metastases in the lungs and the bones of immuno-deficient mice as a model of highly metastatic cancer cells selected in vivo from a single parental cell line and thus with nearly identical genetic background [23,24]. The non-metastatic cell line MCF-7 was used for comparison. In this model system, we show a clear correlation between CerK expression, activity, and metastatic potential of the cells, and we demonstrate that CerK substantially contributes to breast cancer migration and invasion by activation of the PI3K/Akt and the Rho kinase pathways.

## 2. Results

C1P contributes to cancer cell proliferation and migration, but the underlying mechanism is still unclear. Here, we investigated the involvement of CerK in the migration and invasion of breast cancer cells in vitro using the breast cancer cell line MDA-MB-231 and two sublines thereof derived from either lung metastasis (4175) or bone metastasis (1833) in immuno-deficient mice [23,24].

**Figure 1:**
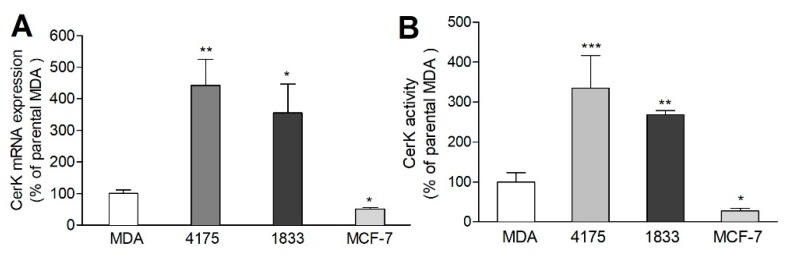
Ceramide kinase (CerK) mRNA expression and activity in parental and metastatic MDA-MB-231 cells and in MCF-7 cells. (**A**) Confluent cells, either parental MDA-MB-231 (MDA), the lung metastatic subline 4175, or the bone-metastatic subline 1833, and MCF-7 were taken for RNA extraction and quantitative PCR analysis of CerK and 18S RNA. (**B**) Confluent cells were incubated for 3 h with 5 μM of C6-NBD-ceramide. Lipids were then extracted, separated by TLC, and analyzed as described in the Methods section. Results are expressed as percentage of parental MDA-MB-231 cells and are means ± SD (*n* = 4), * *p* < 0.05, ** *p* < 0.01, *** *p* < 0.001 considered statistically significant compared to the parental MDA-MB-231 values.

In a first step, CerK mRNA expression was analyzed in the different cell lines. As shown in Figure 1A, CerK mRNA was several-fold upregulated in lung- and bone-derived sublines compared to the parental cells. In addition, cellular CerK activity was also increased in the two sublines (Figure 1B), suggesting CerK to contribute to the metastatic phenotype of the cells. For comparison, we also examined the non-metastatic MCF-7 cell line [25]. Indeed, MCF-7 cells showed much lower CerK mRNA (Figure 1A) and CerK activity (Figure 1B), thus further supporting the hypothesis that CerK expression correlates with the metastatic potential of cancer cells. Unfortunately, since the commercially available CerK antibody ab38011 from Abcam failed to recognize its expected antigen, we were unable to confirm changes of CerK on the protein level (Figure S1).

**Figure 2:**
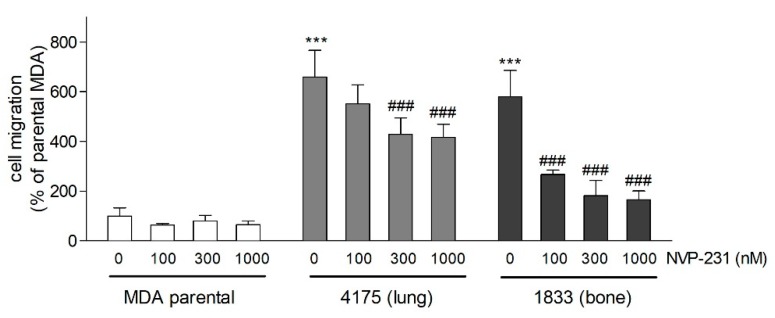
Effect of the CerK inhibitor NVP-231 on cell migration of parental and metastatic MDA-MB-231 cells. 5 × 10^4^ parental MDA-MB-231 cells (open columns), lung metastatic (4175, light grey columns), and bone metastatic (1833, dark grey columns) cells were seeded onto transwell filters and treated for 20 h with either vehicle (0) or the indicated concentrations of the CerK inhibitor NVP-231 in Dulbecco’s Modified Eagle Medium (DMEM)/1% fetal bovine serum (FBS). Migrated cells were determined as described in the Methods section. Representative pictures are shown in Supplementary Figure S2. Data are expressed as percentage of control parental MDA-MB-231 cells migrated into the lower chamber and are the means ± SD *(n* = 3). *** *p* < 0.001 compared to vehicle-treated parental MDA-MB-231 cells; ^###^ *p* < 0.001 compared to the vehicle-treated 4175 or 1833 cells.

The migratory capacity of cells was measured in an adapted Boyden chamber assay. Both metastatic cell lines showed enhanced migration compared to the parental cells (Figure 2), which confirms previous findings in this metastases model [26]. As expected, in the presence of the CerK inhibitor NVP-231 [27], migration of the two sublines dose-dependently decreased (Figure 2), reaching maximal inhibition of 30% at 1 μM in 4175 cells and of 70% at 1 μM in 1833 cells. At this concentration, cell viability was not affected. NVP-231 was confirmed as a potent CerK inhibitor in a cellular activity assay showing an almost complete inhibition at 1 μM in all cell lines (Figure S3).

**Figure 3:**
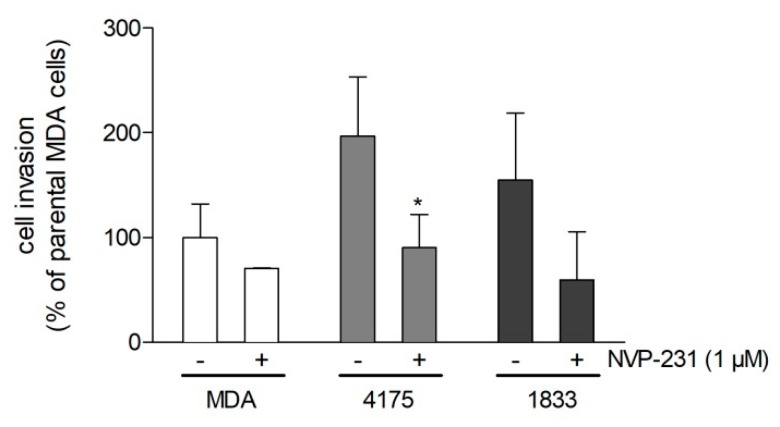
Effect of the CerK inhibitor NVP-231 on cell invasion of parental and metastatic MDA-MB-231 cells. 5 × 10^4^ parental MDA-MB-231 cells (open columns), lung metastatic (4175, grey columns), and bone metastatic (1833, black columns) cells were seeded onto Matrigel-coated transwell filters and treated for 48 h in the absence (−) or the presence (+) of NVP-231 (1 μM) in DMEM/1% FBS. Invaded cells were determined as described in the Methods section. Representative images are shown in Supplementary Figure S4. Data are expressed as percentage of parental MDA cells and are means ± SD (*n* = 3). * *p* < 0.05 compared to vehicle-treated 4175 cells.

Another feature of metastatic cells is invasiveness [28,29]. We previously reported that the 4175 and the 1833 sublines also have an increased capacity of invasion, as detected in a Matrigel assay [26]. Here, we found that this process was also mitigated by the CerK inhibitor NVP-231 (Figure 3).

**Figure 4:**
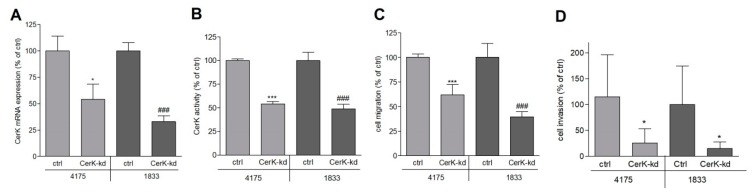
Effect of CerK knockdown on cell migration of metastatic MDA-MB-231 cells. (**A**,**B**) Lung metastatic (4175) and bone metastatic (1833) MDA-MB-231 cells that were stably transduced with a lentiviral construct containing CerK small hairpin RNA (shRNA) (CerK-kd) or a control construct (ctrl) were analyzed for CerK mRNA expression (**A**) and CerK activity (**B**), as described in the Methods section. (**C**) 5 × 10^4^ control cells (ctrl) or the CerK-kd 4175 and 1833 cells were seeded onto transwell filters and allowed to migrate for 20 h in DMEM containing 1% FBS. (**D**) 5 × 10^4^ control cells (ctrl) or the CerK-kd 4175 and 1833 cells were seeded onto Matrigel-precoated transwell filters, as described in the Methods section, and incubated for 24 h in growth medium to allow for invasion. Migrated and invaded cells were quantified as described in the Methods section. Representative pictures are shown in Supplementary Figures S5 and S6. Data are expressed as percentage of ctrl cells and are means ± SD (*n* = 3 for **A**–**C**, *n* = 6 for **D**). * *p* < 0.05, *** *p* < 0.001 compared to 4175 ctrl cells; ^###^ *p* < 0.001 compared to 1833 ctrl cells.

To verify that the anti-migratory effect of pharmacological inhibition of CerK can be reproduced by a genetic approach, we stably downregulated CerK expression in the 4175 and the 1833 sublines by lentiviral transduction using a CerK-directed small hairpin RNA (shRNA) construct. After selection of stable clones, we found that downregulation efficiency on the mRNA level was 46% for 4175 cells and 67% for 1833 cells (Figure 4A); consequently, cellular CerK activity was decreased by 46% in 4175 and 51% in 1833 cells (Figure 4B). Importantly, the CerK downregulated cells migrated and invaded much less compared to control cells transduced with the empty lentiviral vector (Figure 4C,D).

**Figure 5:**
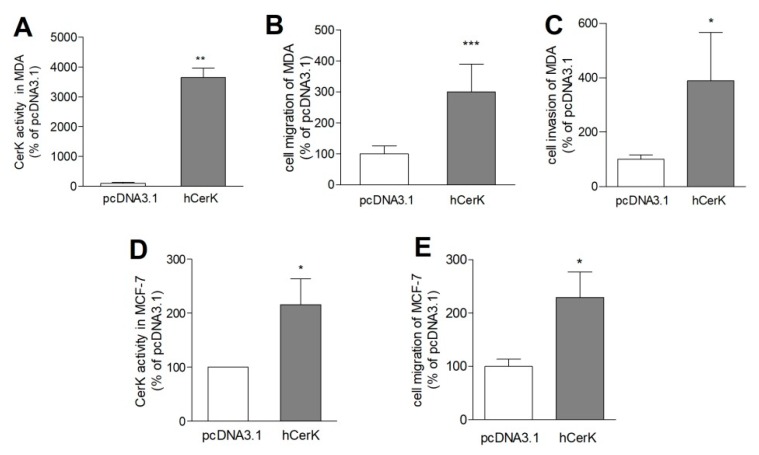
Effect of CerK overexpression on cell migration of parental MDA-MB-231 cells and MCF-7 cells. MDA-MB-231 cells (**A**–**C**) or MCF-7 cells (**D**,**E**) were transiently transfected with either an empty vector (pcDNA3.1) or a vector containing the human CerK cDNA (hCerK). 48 h after transfection, cells were examined in a CerK activity assay (**A**,**D**), in a cell migration assay (**B**,**E**), or in an invasion assay (**C**), as described in the Methods section. Representative pictures of invaded cells are shown in Supplementary Figure S7. Data are expressed as percentage of pcDNA3.1 controls and are means ± SD (*n* = 3). * *p* < 0.05, ** *p* < 0.01, *** *p* < 0.001 compared to pcDNA3.1-treated control cells.

Next, we used the opposite approach of overexpressing CerK in the parental MDA-MB-231 cell line. As shown in Figure 5, in these cells, CerK activity increased several-fold (Figure 5A) and, consequently, cells migrated and invaded significantly more than transfected control cells (Figure 5B,C). Similarly, overexpression of CerK also increased CerK activity (Figure 5D) and the migration of MCF-7 cells (Figure 5E), albeit their basal migratory capacity was much lower compared to MDA-MB-231 cells. No invasion was detected in MCF-7 cells under any condition.

**Figure 6:**
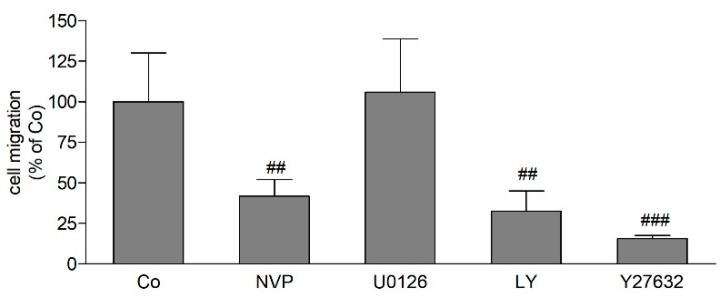
Effect of CerK, MEK, phosphoinositide 3-kinase (PI3K), and RhoA-dependent protein kinase (ROCK) inhibition on migration of CerK-overexpressed parental MDA-MB-231 cells. 5 × 10^4^ parental MDA-MB-231 cells transfected with the human CerK cDNA in a pcDNA3.1 vector (CerKoe) cells were seeded onto transwell filters and incubated for 20 h in DMEM/1% FBS in the absence (Co) or the presence of the CerK inhibitor NVP-231 (NVP, 1 μM), the MEK inhibitor U0126 (10 μM), the PI3K inhibitor LY294002 (LY, 10 μM), and the RhoA-dependent protein kinase (ROCK) inhibitor Y27632 (10 μM). Migrated cells were determined as described in the Methods section. Data are expressed as % of CerKoe control cells and are means ± SD (*n* = 3), ^##^ *p* < 0.01, ^###^ *p* < 0.001 considered statistically significant compared to the CerKoe control values.

We further treated CerK overexpressing MDA-MB-231 cells with a panel of inhibitors of selected molecular targets to unveil possible signaling pathways involved in migration. As expected, the CerK inhibitor mitigated the enhanced migration resulting from CerK overexpression (Figure 6). Moreover, inhibition of PI 3-kinase signaling with LY294002 as well as inhibition of the RhoA-dependent protein kinase (ROCK) by Y27632 also attenuated migration, whereas the MEK inhibitor U0126 was ineffective (Figure 6). All inhibitors were confirmed to block their target and downstream signaling, as shown in Figure S8.

**Figure 7:**
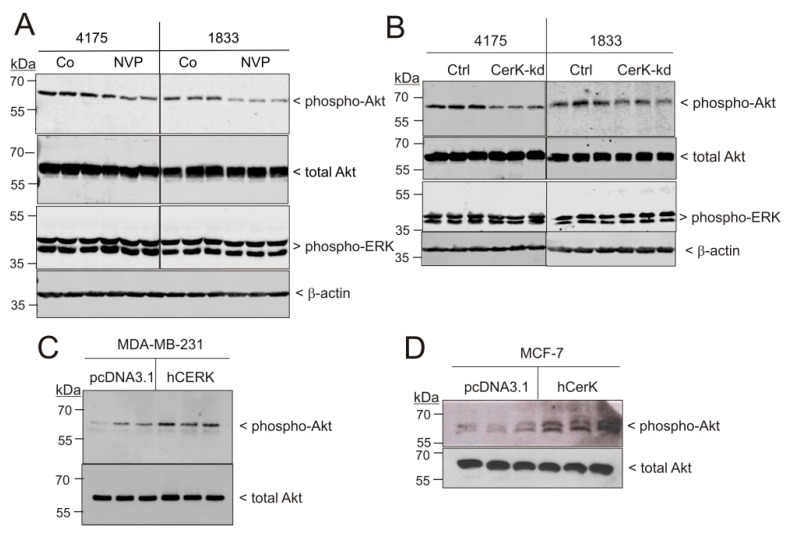
Effect of CerK inhibition, downregulation, and overexpression on phosphorylation of Akt in MDA-MB-231 and MCF-7 cells. (**A**) Confluent 4175 and 1833 cells were incubated for 24 h with either vehicle (Co) or NVP-231 (NVP, 1 μM). (**B**) Then, 4175 and 1833 cells transduced with either a control vector (Ctrl) or a CerK shRNA construct (CerK-kd) were incubated for 24 h in serum-free DMEM. (**C**,**D**) Parental MDA-MB-231 cells (**C**) and MCF-7 cells (**D**) were transiently transfected with an empty vector (pcDNA3.1) or a vector containing the human CerK cDNA (hCerK) for overexpression. Confluent cells were incubated for 20 h in serum-free DMEM. Thereafter, protein lysates were homogenized and separated by SDS-PAGE, transferred to nitrocellulose, and subjected to Western blotting using antibodies against phospho-Ser^473^-Akt, total Akt, phospho-ERK1/2, and β-actin. Results show triplicates of one representative experiment from at least three independent determinations. Bands corresponding to phospho-Akt and total Akt were densitometrically evaluated and are depicted in Supplementary Figure S9.

On the molecular level, treatment of the metastatic sublines 4175 and 1833 with NVP-231 decreased the level of phospho-Akt (Figure 7A), and reduced phospho-Akt was also seen in CerK-kd cells (Figure 7B). No change of phospho-ERK1/2 was seen (Figure 5A,B). On the contrary, overexpression of CerK in parental MDA-MB-231 cells (Figure 7C) and in MCF-7 cells (Figure 7D) increased Akt phosphorylation in both cell types. This supports the conclusion that CerK activates PI3K/Akt signaling, which is crucial for the here reported migration and invasion of metastatic breast cancer cells.

## 3. Discussion

CerK converts pro-apoptotic ceramide to C1P, which regulates pro-malignant cell responses such as survival, proliferation, migration, and invasion [8,30]. Previously, it was shown that CerK expression is higher in estrogen receptor (ER) negative than in ER positive breast cancer tissue, and that within the ER negative subgroup of patients, those with highest CerK expression had the worst prognosis and the shortest survival [21]. Additionally, CerK correlates with a higher risk of tumor recurrence in women with breast cancer [22]. Here, we focused on differences in CerK expression and activity between high and non-metastatic breast cancer cell lines and investigated the effect of genetic and pharmacologic CerK modulators on cell motility and invasion in vitro. The cell lines 4175 and 1833 are single-cell derived progenies of the triple-negative, metastatic MDA-MB-231 breast cancer cell line previously isolated from lung and bone metastases of immuno-deficient mice. The use of the sublines, which were selected in vivo to survive as metastases at distant sites, allowed us to compare cells with a very similar genetic background and identical origin and to identify differences implicated in metastasis, such as migration and invasion. The non-metastatic cell line MCF-7 was included in the study for comparison.

We found a good correlation between the level of CerK expression and activation and migratory capacity of the cells, which was higher in 4175 and 1833 than in parental MDA-MB-231 cells and lowest in MCF-7 cells. As expected, genetic knockdown or pharmacological inhibition of CerK with NVP-231 in 4175 and 1833 cells reduced migration and invasion, whereas overexpression of CerK in the parental MDA-MB-231 cells and in MCF-7 cells enhanced their migratory potential. This suggests that CerK is a potent driver of metastasis in breast cancer and is in line with recent findings that CerK positively regulates migration and invasion of pancreatic cancer cells [13].

The pro-malignant features of CerK are still poorly understood. Particularly, the site of action of C1P is disputed as both extracellular action through a still undefined receptor [31], and intracellular action by binding to intracellular targets, including the cytosolic phospholipase A_2_ (cPLA_2_) [32], have been reported. CerK is an intracellular lipid kinase and, consequently, C1P first appears in intracellular compartments. The intracellular target of C1P and how the signal is transduced to affect migration is unknown. In the pancreatic cancer model, exogenous C1P was recently reported to enhance cancer cell migration in a pertussis toxin-dependent manner, suggesting that C1P somehow stimulates a G_i/0_ protein-coupled receptor (GPCR) [13]. Since no high-affinity C1P receptor has so far been identified, and considering that rather high concentrations of C1P were required to see cellular effects, this argues for the existence of a low-affinity receptor or an unspecific effect on another lipid-binding GPCR entity. Furthermore, the authors of the study suggested a role for PI3K, mTOR, and ROCK in the regulation of migration [13]. Using CerK knockdown and overexpression in our breast cancer cell lines, we found that, in addition to PI3K and ROCK, CerK also activates Akt, another major driver of malignancy [33,34].

The cPLA_2_ is another intracellular target of C1P, which, upon C1P binding, is activated to release arachidonic acid and thereby increases prostaglandin E_2_ (PGE_2_) formation [32]. Chronic inflammation is a well-known risk factor for cancer development and, in view of our previous findings that the metastatic sublines 4175 and 1833 produce much higher amounts of PGE_2_ [26] and that cPLA_2_ is implicated in breast cancer cell migration [35,36], it is plausible that the pro-migratory effect of CerK/C1P involves cPLA_2_. However, in our breast cancer model, neither inhibition of CerK with NVP-231 nor CerK knockdown could attenuate PGE_2_ formation (Figure S10), thus excluding the notion that high PGE_2_ production in metastatic cells is due to enhanced C1P production by CerK. We believe that these data are robust, since at concentrations of ≤ 1 μM, NVP-231 is highly specific for CerK [27].

CerK was also shown to stimulate migration and tube formation of murine skin dermal microcapillary endothelial cells [37]. In this experiment, cells isolated from CerK (−/−) mice or from wildtype mice treated with NVP-231 showed impaired tube formation, which could not be rescued by vascular endothelial growth factor, basic fibroblast growth factor, or tumor necrosis factorα. This suggests a new mechanism of migration regulated by CerK, which was also activated independent of S1P receptor signaling [37]. Furthermore, C1P can be released by damaged cells, for example, in ischemic myocardium or in bone marrow upon irradiation, and acts as a chemoattractant to trigger migration of multipotent stromal stem cells to allow tissue/organ regeneration [38]. In the same study, exogenous C1P also stimulated migration and tube formation of a human umbilical vein endothelial cell line and promoted in vivo vascularization of Matrigel implants [38]. From this, the authors concluded the existence of a C1P receptor in the migratory and regenerative process without further investigating its molecular entity.

On the other hand, opposite findings on the role of CerK in cell migration have also been described. For example, CerK was shown to inhibit migration and wound healing using fibroblasts and skin biopsies from CerK (+/+) and CerK (−/−) mice [39]. Moreover, in human tissues of wound healing, C1P levels increased during the early healing stage and correlated with increased proliferation and migration of fibroblasts [39]. Altogether, this contradicts data from renal fibroblasts isolated from CerK deficient mice, as these cells showed strongly reduced proliferation rates compared to their wildtype counterparts [40].

How CerK expression and activity are regulated by pro-migratory stimuli and whether this can explain the conflicting data on the role of CerK in cell migration remains to be investigated in more detail. Thus far, it is known that the proliferator-activated receptor (PPAR)-β/δ can act as a transcription factor stimulating CerK gene transcription, and a PPAR-β/δ antagonist unveiled anti-tumor effects of PPAR-β/δ by attenuating progression and metastasis of melanoma [41]. In addition, in a choriocarcinoma cell line, increase of cAMP by forskolin upregulated CerK through CREB1-dependent promotor activation [42] and also stimulated cell migration and invasion [43]. CerK is also post-translationally modified by phosphorylation on different sites, including S^300^, S^340^, S^408^, and S^427^ [44], and CerK activity was shown to be inhibited by protein kinase C (PKC)-βI/II-mediated phosphorylation [45]. Since PKC-βII is a tumor suppressor, and patients with low levels of PKC-βII have a shorter survival [46], one might speculate that CerK is a substrate of PKC-βII, and its inactivation by phosphorylation facilitates tumor regression. In addition, CerK function depends on the presence of serum and growth factors, and its activity is rapidly depleted upon serum deprivation [47]. More data are required to better understand how CerK is regulated in various pathologic conditions and when CerK targeting might be most promising.

In conclusion, we demonstrate for the first time that CerK expression and activity correlate well with the metastatic potential of breast cancer cells and are enhanced in cells after dissemination to distant sites. Together with our data that CerK is implicated in migration and invasion by activating PI3K and Akt signaling in metastatic cells, this warrants further investigation to better understand the pro-malignant function of CerK and to assess the therapeutic potential of CerK targeting in breast cancer.

## 4. Materials and Methods

### 4.1. Chemicals

C6-NBD-ceramide, NVP-231, LY-294002, KICqStart^®^SYBR^®^Green qPCR ReadyMix™ (SYBRgreen), hCerK MISSION^R^ shRNA glycerol stocks, and the anti-β-actin (clone AC-15) antibody were from Sigma-Aldrich Chemie GmbH (Buchs, Switzerland). U-0126 was from InvivoGen (Nunningen, Switzerland). Y-27632 was from Tocris (Zug, Switzerland). Fluorescently-labeled Odyssee IRdye 800CW secondary antibodies were from LI-COR Biosciences (Bad Homburg, Germany). Primers for qPCR were from Eurofins Genomics Germany GmbH (Ebersberg, Germany). The First Strand DNA Synthesis Kit was from ThermoFisher Scientific (Zug, Switzerland) and RNAsolv^®^ from VWR International AG (Dietikon, Switzerland). Antibodies against phospho-Ser^473^-Akt and total Akt were from Cell Signaling/BioConcept (Allschwil, Switzerland).

### 4.2. Cell Lines and Cell Culture Conditions

The human breast cancer cell line MDA-MB-231 and its metastatic sublines 4175 and 1833, which were derived by single-cell cloning from lung and bone metastases, respectively, of immune-deficient mice were provided by Dr. J. Massagué (Memorial Sloan-Kettering Cancer Center, New York, NY, USA). The human breast cancer cell line MCF-7 (HTB-22) was obtained from the American Type Culture Collection (Manassas, VA, USA). Cells were maintained in Dulbecco’s Modified Eagle Medium (DMEM) supplemented with 10% (v/v) fetal bovine serum (FBS), 10 mM HEPES pH 7.4, 100 units/mL penicillin, and 100 μg/mL streptomycin. All cells were grown at 37 °C in a humidified atmosphere containing 5% CO_2_.

### 4.3. CerK Overexpression in Parental MDA-MB-231 and MCF-7 Cells

For CerK overexpression, parental MDA-MB-231 and MCF-7 cells were transfected with a human CerK pcDNA3.1 construct kindly provided by Dr. Frederic Bornancin (Novartis Institute of Biomedical Research, Basel, Switzerland) [48]. The empty pcDNA3.1 plasmid vector was used as control. Cells were subcultured in 100 mm dishes over night to achieve ~70% confluency on the day of transfection. Then, 6 μg of DNA and 12 μL of Turbofect transfection reagent (Thermo-Fisher) were mixed in 600 μL Opti-MEM^TM^ and added to the cells for 48 h. Stable transfectants were obtained under selection conditions in the presence of 1 mg/mL G418.

### 4.4. CerK Knockdown in the Metastatic Sublines 4175 and 1833

Knockdown of CerK was performed with short hairpin RNA (shRNA). The target sequence of shCerK was: CCGGCACACTGAGTTCTCTATATTTCTCGAGAAATATAGAGAACTCAGTGTGTTTTTG.

shRNA plasmids were virally packaged in HEK-293T cells, which were co-transfected with the retroviral constructs pLP1, pLP2, and pVSV-G according to the manufacturer’s instructions (Invitrogen). Briefly, HEK-293T cells were seeded in a 6 well plate to be ~60% confluent on the day of transfection. Then, 3 μg DNA and 15 μg polyethylenimine (PEI) were mixed in 200 μL 150 mM NaCl and added dropwise to the cells. Supernatant containing viral particles was collected 48 h post-transfection, filtered, and used to infect the metastatic sublines 4175 and 1833. In addition, cells were transduced with Sigma Mission^R^TRC lentiviral particles containing pLKO.5-Puro empty vector as control. Transduction was performed in the presence of 6 μg/mL polybrene. Stable clones were selected by including 1 µg/mL puromycin in the growth medium.

### 4.5. Cell Stimulation, Homogenization, and Western Blotting

Cells were incubated for 24 h in serum-free DMEM prior to stimulation as specified in the figure legends. To stop stimulation, the supernatant was discarded, and the cell monolayer was washed with phosphate-buffered saline (PBS), scraped, and homogenized by sonication in lysis buffer [26]. Samples were centrifuged for 10 min at 13,000× *g*, and 30 μg of protein from the supernatant was separated by SDS–PAGE, transferred to nitrocellulose membrane, and used for Western blot analysis using the antibodies as indicated in the figure legends.

### 4.6. Quantitative Real-Time PCR

Total RNA (1 μg) isolated with RNAsolv^®^ (VWR) reagent was taken for reverse transcriptase PCR and real-time PCR using SYBRgreen according to standard methods. The following primers were used: human CerK: forward CAC CTT AGC CTC CAT CAC CAC TG; reverse AAC ATA CCA TCT CCG CCG ACA C; human 18S: forward CGA TTC CGT GGG TGG TGG TG; reverse CAT GCC AGA GTC TCG TTC GTT ATC. Values were normalized to 18S RNA, and the fold induction was calculated by applying the ΔΔC_T_ method.

### 4.7. CerK Activity Assay

After incubation for 3 h with 5 µM C6-NBD-ceramide in DMEM medium containing 10% FBS, cells were rinsed with PBS, and lipids were extracted with 2 mL methanol, 2 mL chloroform, 100 µL 1 M hydrochloric acid, and 1.6 mL salt solution (0.74% KCl, 0.04% CaCl_2_, 0.034% MgCl_2_). The samples were vortexed and centrifuged, and the organic phase was taken and dried using a vacuum centrifuge. The dried lipid fraction was redissolved in 20 µL methanol and separated by thin layer chromatography (TLC) using Silica gel 60 plates and chloroform/acetone/methanol/acetic acid/water (10:4:3:2:1; vol/vol) as developing solvent. TLC plates were visualized at 532 nm using the Typhoon 9400 scanner (GE Healthcare), and quantitative analysis was performed with the ImageStudio software (LI-COR Biosciences).

### 4.8. Migration Assay

To measure cell migration, an adapted Boyden chamber assay was used as previously described [26]. To this end, cells were incubated for 20 h in serum-free DMEM and then seeded into a transwell filter (6.5 μm diameter, 8 μm pore size) at a density of 5 × 10^4^ cells in 200 μL starvation medium. Then, 600 μL of DMEM medium containing 1% FBS was filled in the lower compartment, and cells were allowed to migrate for 20 h at 37 °C. Migrated cells in the lower chamber were counted in five different random fields per well using a bright field microscope (Zeiss Observer Z1). Alternatively, MCF-7 cells that had migrated into the filter were stained with 4′,6-diamidine-2′-phenylindole dihydrochloride (DAPI) (1 μg/mL in methanol) and quantified in five random fields under a fluorescent microscope (Zeiss Observer Z1).

### 4.9. Invasion Assay

Cell invasion was also measured in an adapted Boyden chamber assay [26] where the transwell filters were precoated with 100 µL of 1 mg/mL Matrigel^R^. Quiescent cells at a density of 5 × 10^4^ cells in 200 µL DMEM were seeded onto the filters, 750 µL of growth medium was added to the lower compartment, and cells were further incubated at 37 °C for 24 h to allow invasion. Thereafter, the transwell filters were removed, and invaded cells were stained for 15 min with DAPI (1 μg/mL in methanol) and quantified in five random fields for one sample under a fluorescent microscope (Zeiss Observer Z1).

### 4.10. Statistical Analysis

For statistical comparison, an unpaired t-test was used when comparing two groups, and one-way ANOVA followed by a Bonferroni post-hoc test were used for multiple comparisons. Software programs used for data analysis were GraphPad Prism 7.02 (GraphPad Software Inc., La Jolla, CA, USA) and Excel 2016 (Microsoft Corporation, Redmond. WA, USA).

## Supplementary Materials

The following are available online at https://www.mdpi.com/1422-0067/21/4/1396/s1.

## Abbreviations

C1P	ceramide 1-phosphate
CerK	ceramide kinase
cPLA_2_	cytosolic phospholipase A_2_
DMEM	Dulbecco’s Modified Eagle Medium
ER	estrogen receptor
FBS	fetal bovine serum
kd	knockdown
PBS	phosphate-buffered saline
PGE_2_	prostaglandin E_2_
PH	pleckstrin homology domain
PI3K	phosphoinositide 3-kinase
PKC	protein kinase C
PPAR	peroxisome proliferator activated receptor
S1P	sphingosine 1-phosphate
SDS-PAGE	sodium dodecyl sulfate-polyacrylamide gel electrophoresis
shRNA	small hairpin RNA
TLC	thin layer chromatography

## References

[1] Bottos A., Hynes N.E. (2014). Cancer staying together on the road to metastasis. Nature.

[2] Siegel R., Naishadham D., Jemal A. (2013). Cancer statistics, 2013. CA-Cancer J. Clin..

[3] Newton J., Lima S., Maceyka M., Spiegel S. (2015). Revisiting the sphingolipid rheostat: Evolving concepts in cancer therapy. Exp. Cell Res..

[4] Huwiler A., Pfeilschifter J. (2006). Altering the sphingosine-1-phosphate/ceramide balance: A promising approach for tumor therapy. Curr. Pharm. Des..

[5] Huwiler A., Zangemeister-Wittke U. (2007). Targeting the conversion of ceramide to sphingosine 1-phosphate as a novel strategy for cancer therapy. Crit. Rev. Oncol. Hematol..

[6] Pyne N.J., Pyne S. (2010). Sphingosine 1-phosphate and cancer. Nat. Rev. Cancer.

[7] Haass N.K., Nassif N., McGowan E.M. (2015). Switching the sphingolipid rheostat in the treatment of diabetes and cancer comorbidity from a problem to an advantage. Biomed. Res. Int..

[8] Presa N., Gomez-Larrauri A., Rivera I.G., Ordonez M., Trueba M., Gomez-Munoz A. (2016). Regulation of cell migration and inflammation by ceramide 1-phosphate. Biochim. Biophys. Acta.

[9] Bornancin F. (2011). Ceramide kinase: The first decade. Cell Signal..

[10] Bajjalieh S.M., Martin T.F., Floor E. (1989). Synaptic vesicle ceramide kinase. A calcium-stimulated lipid kinase that co-purifies with brain synaptic vesicles. J. Biol. Chem..

[11] Hinkovska-Galcheva V.T., Boxer L.A., Mansfield P.J., Harsh D., Blackwood A., Shayman J.A. (1998). The formation of ceramide-1-phosphate during neutrophil phagocytosis and its role in liposome fusion. J. Biol. Chem..

[12] Mitsutake S., Kim T.J., Inagaki Y., Kato M., Yamashita T., Igarashi Y. (2004). Ceramide kinase is a mediator of calcium-dependent degranulation in mast cells. J. Biol. Chem..

[13] Rivera I.G., Ordonez M., Presa N., Gangoiti P., Gomez-Larrauri A., Trueba M., Fox T., Kester M., Gomez-Munoz A. (2016). Ceramide 1-phosphate regulates cell migration and invasion of human pancreatic cancer cells. Biochem. Pharmacol..

[14] Pastukhov O., Schwalm S., Zangemeister-Wittke U., Fabbro D., Bornancin F., Japtok L., Kleuser B., Pfeilschifter J., Huwiler A. (2014). The ceramide kinase inhibitor NVP-231 inhibits breast and lung cancer cell proliferation by inducing M phase arrest and subsequent cell death. Br. J. Pharmacol..

[15] Pettus B.J., Bielawska A., Spiegel S., Roddy P., Hannun Y.A., Chalfant C.E. (2003). Ceramide kinase mediates cytokine- and calcium ionophore-induced arachidonic acid release. J. Biol. Chem..

[16] Sugiura M., Kono K., Liu H., Shimizugawa T., Minekura H., Spiegel S., Kohama T. (2002). Ceramide kinase, a novel lipid kinase. Molecular cloning and functional characterization. J. Biol. Chem..

[17] Rovina P., Schanzer A., Graf C., Mechtcheriakova D., Jaritz M., Bornancin F. (2009). Subcellular localization of ceramide kinase and ceramide kinase-like protein requires interplay of their pleckstrin homology domain-containing N-terminal regions together with C-terminal domains. Biochim. Biophys. Acta.

[18] Kim T.J., Mitsutake S., Igarashi Y. (2006). The interaction between the pleckstrin homology domain of ceramide kinase and phosphatidylinositol 4,5-bisphosphate regulates the plasma membrane targeting and ceramide 1-phosphate levels. Biochem. Biophys. Res. Commun..

[19] Lamour N.F., Stahelin R.V., Wijesinghe D.S., Maceyka M., Wang E., Allegood J.C., Merrill A.H., Cho W., Chalfant C.E. (2007). Ceramide kinase uses ceramide provided by ceramide transport protein: Localization to organelles of eicosanoid synthesis. J. Lipid Res..

[20] Mishra S.K., Gao Y.G., Deng Y., Chalfant C.E., Hinchcliffe E.H., Brown R.E. (2018). Cptp: A sphingolipid transfer protein that regulates autophagy and inflammasome activation. Autophagy.

[21] Ruckhaberle E., Karn T., Rody A., Hanker L., Gatje R., Metzler D., Holtrich U., Kaufmann M. (2009). Gene expression of ceramide kinase, galactosyl ceramide synthase and ganglioside GD3 synthase is associated with prognosis in breast cancer. J. Cancer Res. Clin. Oncol..

[22] Payne A.W., Pant D.K., Pan T.C., Chodosh L.A. (2014). Ceramide kinase promotes tumor cell survival and mammary tumor recurrence. Cancer Res..

[23] Minn A.J., Gupta G.P., Siegel P.M., Bos P.D., Shu W.P., Giri D.D., Viale A., Olshen A.B., Gerald W.L., Massague J. (2005). Genes that mediate breast cancer metastasis to lung. Nature.

[24] Kang Y.B., Siegel P.M., Shu W.P., Drobnjak M., Kakonen S.M., Cordon-Cardo C., Guise T.A., Massague J. (2003). A multigenic program mediating breast cancer metastasis to bone. Cancer Cell.

[25] Bozzuto G., Condello M., Molinari A. (2015). Migratory behaviour of tumour cells: A scanning electron microscopy study. Annali dell’Istituto Superiore di Sanita.

[26] Filipenko I., Schwalm S., Reali L., Pfeilschifter J., Fabbro D., Huwiler A., Zangemeister-Wittke U. (2016). Upregulation of the S1P3 receptor in metastatic breast cancer cells increases migration and invasion by induction of PGE2 and EP2/EP4 activation. Biochim. Biophys. Acta.

[27] Graf C., Klumpp M., Habig M., Rovina P., Billich A., Baumruker T., Oberhauser B., Bornancin F. (2008). Targeting ceramide metabolism with a potent and specific ceramide kinase inhibitor. Mol. Pharmacol..

[28] Yang Y., Zheng H.M., Zhan Y.T., Fan S.Q. (2019). An emerging tumor invasion mechanism about the collective cell migration. Am. J. Transl. Res..

[29] Yilmaz M., Christofori G. (2010). Mechanisms of motility in metastasizing cells. Mol. Cancer Res..

[30] Hait N.C., Maiti A. (2017). The role of sphingosine-1-phosphate and ceramide-1-phosphate in inflammation and cancer. Mediators Inflamm..

[31] Granado M.H., Gangoiti P., Ouro A., Arana L., Gonzalez M., Trueba M., Gomez-Munoz A. (2009). Ceramide 1-phosphate (C1P) promotes cell migration involvement of a specific C1P receptor. Cell Signal..

[32] Stahelin R.V., Subramanian P., Vora M., Cho W., Chalfant C.E. (2007). Ceramide-1-phosphate binds group iva cytosolic phospholipase A2 via a novel site in the C2 domain. J. Biol. Chem..

[33] Lien E.C., Dibble C.C., Toker A. (2017). PI3K signaling in cancer: Beyond Akt. Curr. Opin. Cell Biol..

[34] Brown J.S., Banerji U. (2017). Maximising the potential of Akt inhibitors as anti-cancer treatments. Pharmacol. Ther..

[35] Tian G., Wang X., Zhang F., Geng H., Hou W., Chen L., Guo H., Zhang N. (2011). Downregulation of cPLA2gamma expression inhibits EGF-induced chemotaxis of human breast cancer cells through Akt pathway. Biochem. Biophys. Res. Commun..

[36] Tunset H.M., Feuerherm A.J., Selvik L.M., Johansen B., Moestue S.A. (2019). Cytosolic phospholipase A2 alpha regulates TLR signaling and migration in metastatic 4T1 cells. Int. J. Mol. Sci..

[37] Niwa S., Graf C., Bornancin F. (2009). Ceramide kinase deficiency impairs microendothelial cell angiogenesis in vitro. Microvasc. Res..

[38] Kim C., Schneider G., Abdel-Latif A., Mierzejewska K., Sunkara M., Borkowska S., Ratajczak J., Morris A.J., Kucia M., Ratajczak M.Z. (2013). Ceramide-1-phosphate regulates migration of multipotent stromal cells and endothelial progenitor cells-implications for tissue regeneration. Stem Cells.

[39] Wijesinghe D.S., Brentnall M., Mietla J.A., Hoeferlin L.A., Diegelmann R.F., Boise L.H., Chalfant C.E. (2014). Ceramide kinase is required for a normal eicosanoid response and the subsequent orderly migration of fibroblasts. J. Lipid Res..

[40] Pastukhov O., Schwalm S., Romer I., Zangemeister-Wittke U., Pfeilschifter J., Huwiler A. (2014). Ceramide kinase contributes to proliferation but not to prostaglandin E2 formation in renal mesangial cells and fibroblasts. Cell Physiol. Biochem..

[41] Tsuji K., Mitsutake S., Yokose U., Sugiura M., Kohama T., Igarashi Y. (2008). Role of ceramide kinase in peroxisome proliferator-activated receptor beta-induced cell survival of mouse keratinocytes. FEBS J..

[42] Euskirchen G., Royce T.E., Bertone P., Martone R., Rinn J.L., Nelson F.K., Sayward F., Luscombe N.M., Miller P., Gerstein M. (2004). CREB binds to multiple loci on human chromosome 22. Mol. Cell Biol..

[43] Zygmunt M., Hahn D., Munstedt K., Bischof P., Lang U. (1998). Invasion of cytotrophoblastic JEG-3 cells is stimulated by HCG in vitro. Placenta.

[44] Chen W.Q., Graf C., Zimmel D., Rovina P., Krapfenbauer K., Jaritz M., Parker P.J., Lubec G., Bornancin F. (2010). Ceramide kinase profiling by mass spectrometry reveals a conserved phosphorylation pattern downstream of the catalytic site. J. Proteome Res..

[45] Takahashi H., Ashikawal H., Nakamura H., Murayama T. (2019). Phosphorylation and inhibition of ceramide kinase by protein kinase C-beta: Their changes by serine residue mutations. Cell Signal..

[46] Dowling C.M., Phelan J., Callender J.A., Cathcart M.C., Mehigan B., McCormick P., Dalton T., Coffey J.C., Newton A.C., O’Sullivan J. (2016). Protein kinase C beta II suppresses colorectal cancer by regulating IGF-1 mediated cell survival. Oncotarget.

[47] Boath A., Graf C., Lidome E., Ullrich T., Nussbaumer P., Bornancin F. (2008). Regulation and traffic of ceramide 1-phosphate produced by ceramide kinase - comparative analysis to glucosylceramide and sphingomyelin. J. Biol. Chem..

[48] Carre A., Graf C., Stora S., Mechtcheriakova D., Csonga R., Urtz N., Billich A., Baumruker T., Bornancin F. (2004). Ceramide kinase targeting and activity determined by its N-terminal pleckstrin homology domain. Biochem. Biophys. Res. Commun..

