# Supplementary material for: Ceramide Kinase Is Upregulated in Metastatic Breast Cancer Cells and Contributes to Migration and Invasion by Activation of PI 3-Kinase and Akt
Source: Int J Mol Sci. 2020 Feb 19;21(4):1396. doi: 10.3390/ijms21041396 (PMC7073039; doi:10.3390/ijms21041396)
Supplement: Supplementary file 1 [file ijms-21-01396-s001.pdf]

Ceramide kinase is upregulated in metastatic breast cancer cells and contributes to migration and invasion by activation of PI 3-kinase and Akt.

Schwalm, Erhardt, Römer, Pfeilschifter, Zangemeister-Wittke and Huwiler

Correspondence: huwiler@pki.unibe.ch

Supplementary data:

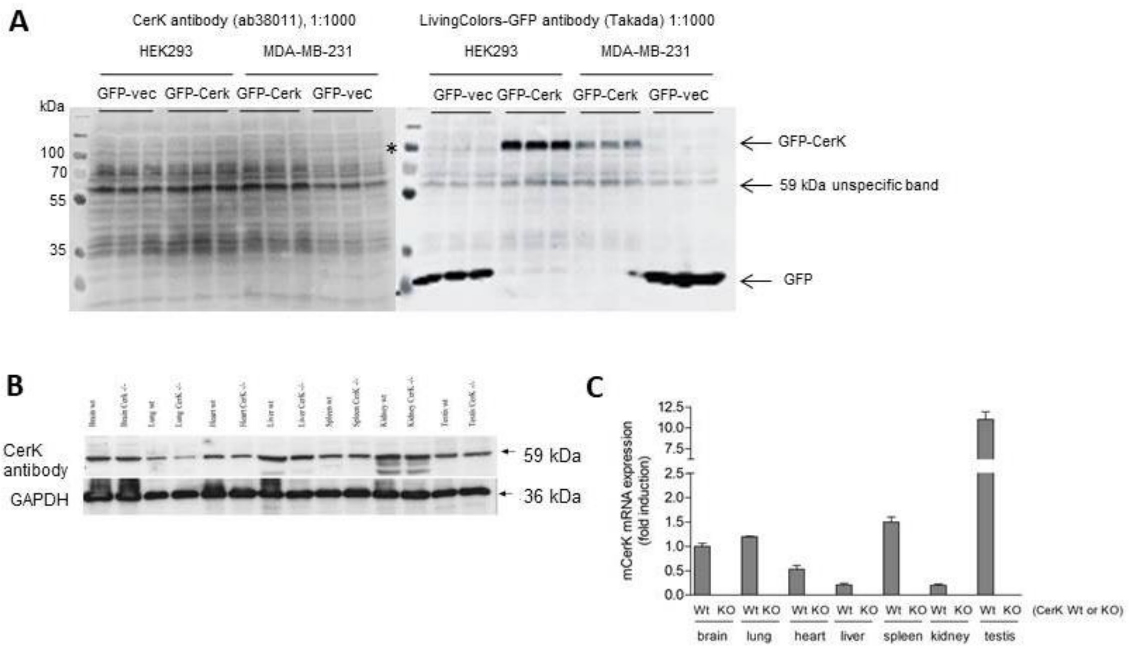

Suppl. Fig. S1

Negative validation of a commercial CerK antibody (ab38011).

(A)HEK293 cells or MDA-MB-2341 cells were transiently transfected with either an empty GFP-vector (GFP-vec) or a GFP-CerK construct. Protein lysates were separated by SDS-PAGE, transferred to nitrocellulose and taken for western blot analysis using either a CerK antibody (ab38011, Abcam, 1:1000) (left panel), or a LivingColor-GFP antibody (Takada, 1:1000) (right panel). (B and C) Protein (B) and RNA (C) extracts of various organs of wildtype (Wt) or CerK knockout (CerK(-/-), KO) mice were prepared and taken for either Western blotting using the CerK antibody (B) or qPCR (C) using primers of mCerK.

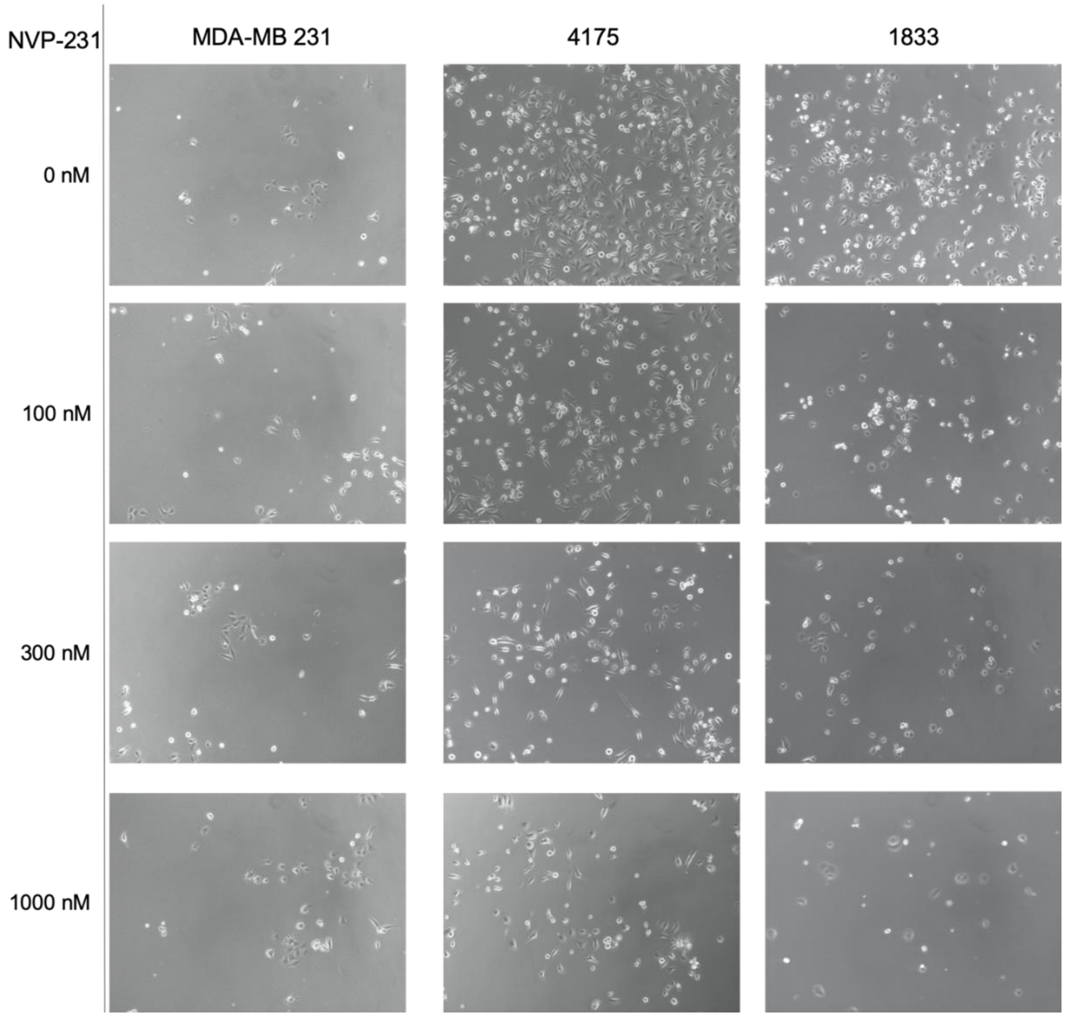

Suppl Fig. S2

Representative light microscopy images of the effect of the inhibitor NVP-231 on migration of parental and metastatic MDA-MB-231 cells.

5×10^4 parental MDA-MB-231 cells, lung metastatic (4175) and bone metastatic (1833) cells were seeded onto transwell filters and treated for 20 h with either vehicle (0 nM) or the indicated concentrations (in nM) of the inhibitor NVP-231. Light microscopic pictures were taken of cells, that migrated through the transwell membrane into the lower chamber (10x magnification, Zeiss Observer Z1).

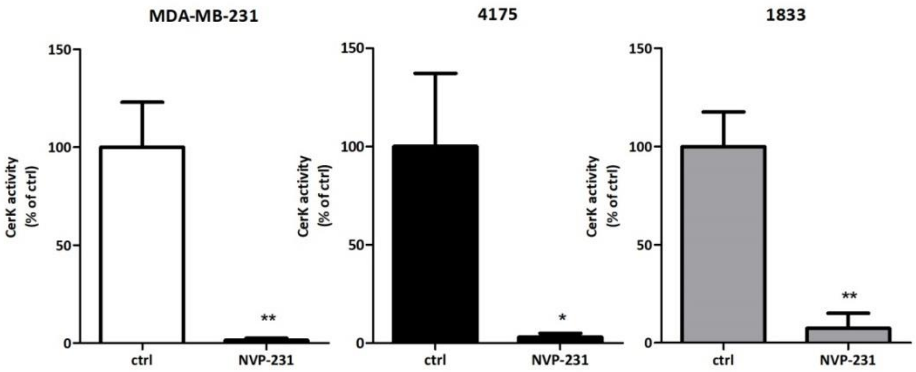

Suppl Fig. S3

Effect of the inhibitor NVP-231 on CerK activity in parental and metastatic MDA-MB-231 cells.

After treatment with 1 µM of NVP-231 for 24 h, cells were incubated with 5 µM of C6-NBD-ceramide for 3 h. Lipids were then extracted, separated by TLC and analysed as described in the Methods section. Results are expressed as % of parental MDA-MB-231 cells and are means ± SD (n=3), *p< 0.05, **p< 0.01 considered statistically significant compared to the ctrl values.

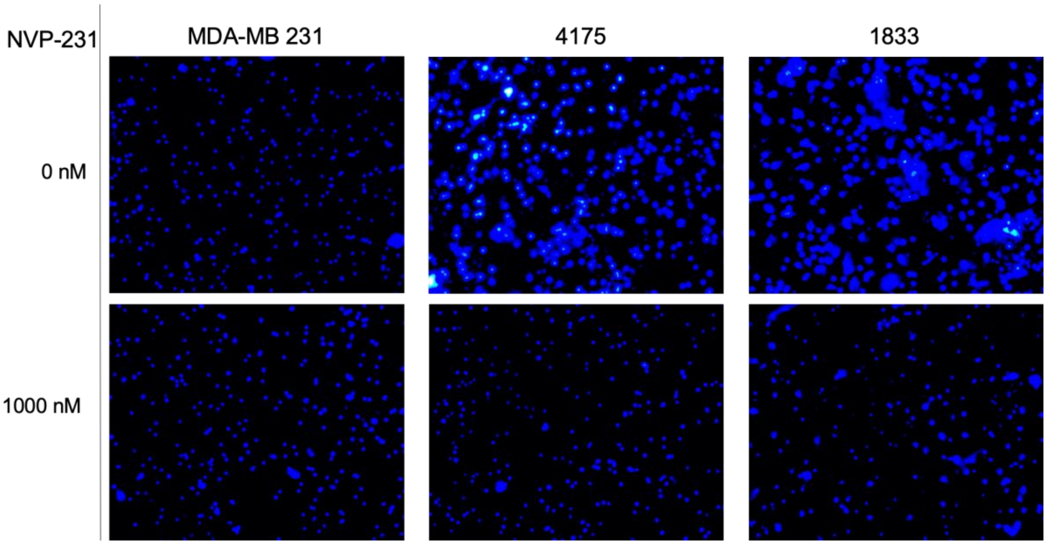

Suppl Fig. S4

Representative fluorescent images of the effect of the inhibitor NVP-231 on invasion of parental and metastatic MDA-MB-231 cells.

Cells were seeded at a density of 2,5 x 10^5 onto Matrigel-precoated transwell filters as described in the Methods section, and incubated for 48 h with either vehicle (0 nM) or 1000 nM of NVP-231 in growth medium to allow invasion. Thereafter, the transwell filters were removed and invaded cells were stained for 15 min with DAPI (1 µg/ml in methanol), and quantified in five random fields for one sample under a fluorescent microscope (Zeiss Observer Z1).

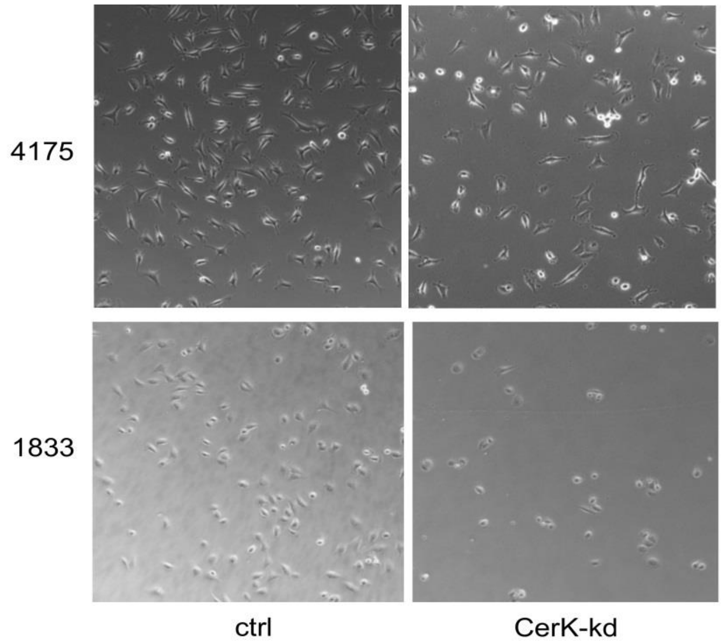

Suppl Fig S5

Representative light microscopy images of the effect of CerK-kd on migration of metastatic 4175 and 1833 cells.

5 × 10^4 control cells (ctrl) or CerK-kd 4175 and 1833 cells were seeded onto transwell filters and allowed to migrate for 20 h in DMEM containing 1 % FBS. Light microscopic pictures were taken of cells, that migrated through the transwell membrane into the lower chamber (10x magnification, Zeiss Observer Z1).

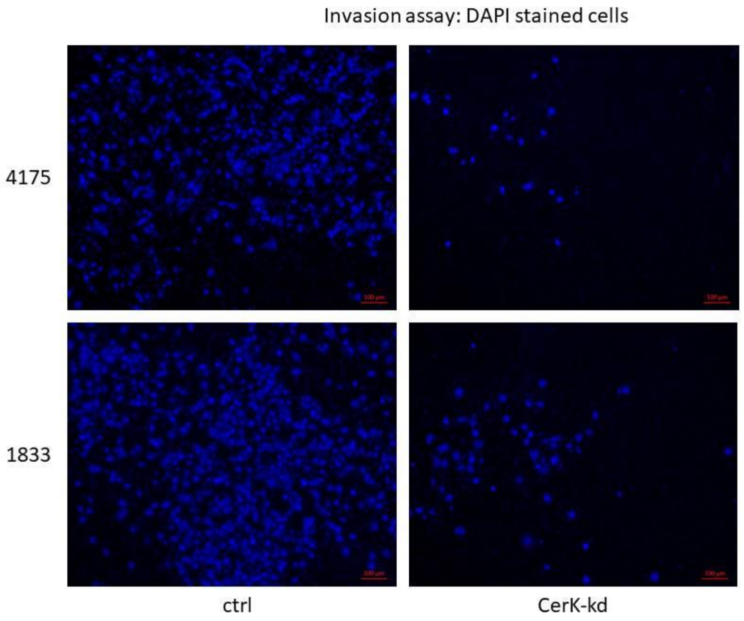

Suppl Fig. S6

Representative fluorescent images of the effect of CerK-kd on invasion of metastatic 4175 and 1833 cells.

5 x 10^4 of cells were seeded onto Matrigel-precoated transwell filters as described in the Methods section, and incubated for 24 h in growth medium to allow invasion. Thereafter, the transwell filters were removed and invaded cells were stained for 15 min with DAPI (1 µg/ml in methanol), and quantified in five random fields for one sample under a fluorescent microscope (Zeiss Observer Z1).

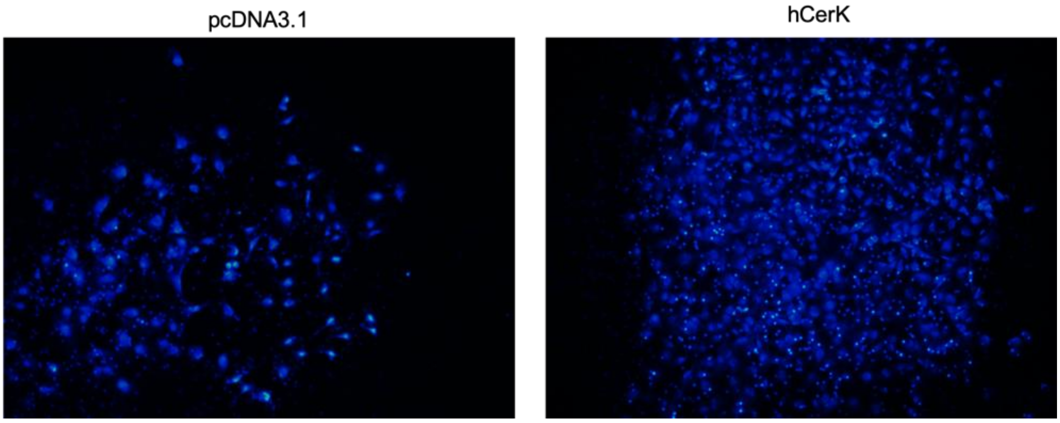

Suppl Fig. S7

Representative fluorescent images of the effect of hCerK overexpression on invasion of parental MDA-MB-231 cells.

5 x 10^4 of cells were seeded onto Matrigel-precoated transwell filters as described in the Methods section, and incubated for 24 h in growth medium to allow invasion. Thereafter, the transwell filters were removed and invaded cells were stained for 15 min with DAPI (1 µg/ml in methanol), and quantified in five random fields for one sample under a fluorescent microscope (Zeiss Observer Z1).

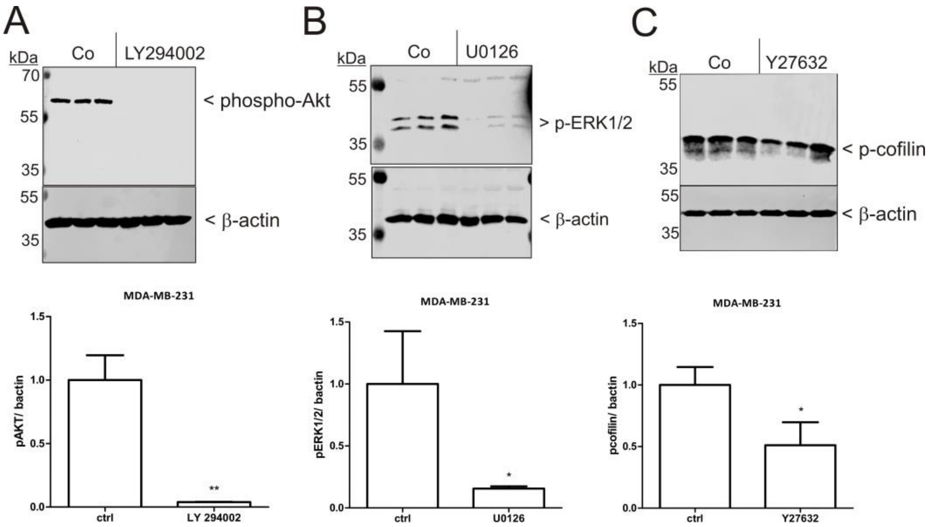

Suppl. Fig S8
Confluent MDA-MB-231 cells were incubated for 20 h in serum-free DMEM and then treated for 24 h with either vehicle (Co, Ctrl) or 10 µM LY294002 (A), 10 µM U0126 (B) or 10 µM Y27632 (C). Thereafter, protein lysates were homogenized and separated by SDS-PAGE, transferred to nitrocellulose and subjected to Western blotting using antibodies against phospho-Ser^{473}-Akt Akt (A), phospho-ERK1/2 (B), or phospho-cofilin (C), and β-actin as a house-keeping protein. Results show triplicates of one representative experiment from at least three independent determinations. Bands corresponding to phospho-ERK1/2, phospho-Akt and phospho-cofilin were densitometrically evaluated. Data are means ± SD (n=3). *p<0.05 compared to MDA-MB-231 ctrl cells.

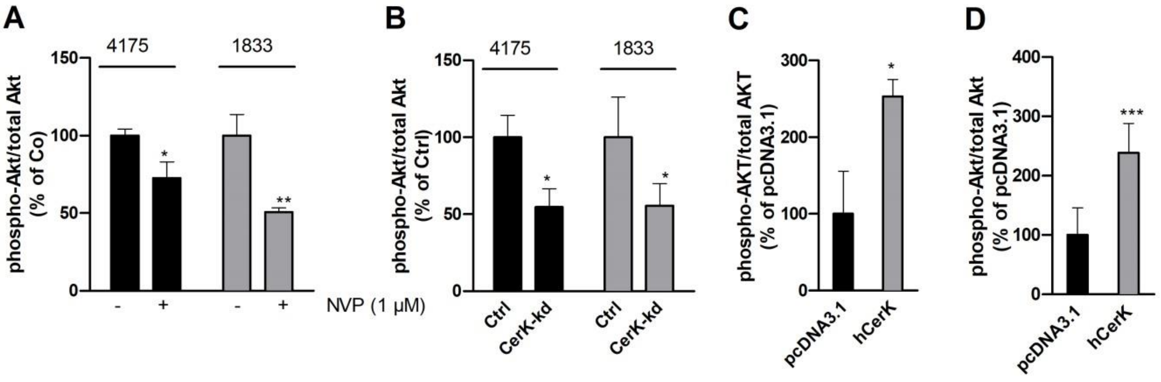

Suppl. Fig S9

Bands corresponding to phospho-Akt and total Akt were densitometrically evaluated by ImageJ software. Data are expressed as means ± S.D. (n=3). *p< 0.05, **p< 0.01, ***p< 0.001 compared to the respective control values.

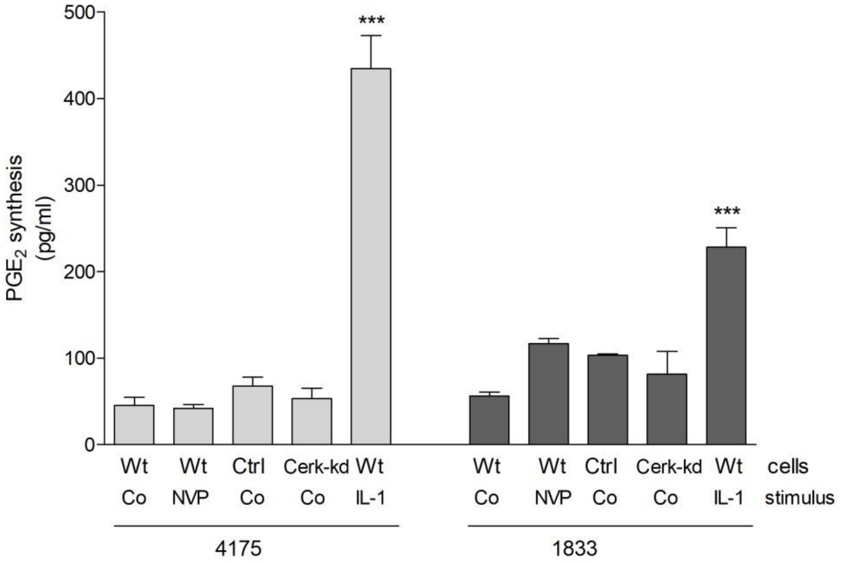

Suppl Fig. S10:

Effect of NVP-231 and Cerk-kd on PGE_2 production in metastatic 4175 and 1833 cells.

Cells in 24-well-plates were treated with vehicle (Co), NVP-231 (1 µM) or IL-1β (1 nM) for 24 h. Untransfected cells (wt) and cells stably transduced with either an empty lentiviral vector (Ctrl) or a vector containing a shRNA against CerK (CerK-kd) were incubated for 24 h in DMEM. Thereafter, supernatants were collected and taken for PGE_2 quantification using an ELISA kit according to the manufacturer's instructions (Enzo Life Sciences, Lörrach, Germany). Data are expressed as pg/ml PGE_2 in the supernatant and are means +/- S.D. (n=3).
